# Single-cell TCRseq: paired recovery of entire T-cell alpha and beta chain transcripts in T-cell receptors from single-cell RNAseq

**DOI:** 10.1186/s13073-016-0335-7

**Published:** 2016-07-27

**Authors:** David Redmond, Asaf Poran, Olivier Elemento

**Affiliations:** 1 Tri-Institutional Training Program in Computational Biology and Medicine, New York, NY USA; 2 Institute for Computational Biomedicine & Institute for Precision Medicine, Weill Cornell Medicine, New York, NY USA

## Abstract

Accurate characterization of the repertoire of the T-cell receptor (TCR) alpha and beta chains is critical to understanding adaptive immunity. Such characterization has many applications across such fields as vaccine development and response, clone-tracking in cancer, and immunotherapy. Here we present a new methodology called single-cell TCRseq (scTCRseq) for the identification and assembly of full-length rearranged V(D)J T-cell receptor sequences from paired-end single-cell RNA sequencing reads. The method allows accurate identification of the V(D)J rearrangements for each individual T-cell and has the novel ability to recover paired alpha and beta segments. Source code is available at https://github.com/ElementoLab/scTCRseq.

**Electronic supplementary material:**

The online version of this article (doi:10.1186/s13073-016-0335-7) contains supplementary material, which is available to authorized users.

## Background

T-cell receptors (TCRs) are molecules found on the outer surface of their corresponding lymphocytes that play a part in recognizing foreign molecules. The generation of diversity in these receptors enabled by somatic recombination involves choosing one from multiple variable (V), diversity (D), and joining (J) to produce VDJ gene segments in TCR beta chains or VJ segments in TCR alpha chains. These receptors play an essential role in regulating the selection, function, and activation of T-cells and also allow the unique identification of a single cell’s clonal ancestry or clonotype. In the case of T-cells, the proper assignment of paired alpha and beta gene rearrangements may also help link T-cell function and TCR specificity [1, 2]. Accurate characterization of these repertoires, including reliable determination of each junction, would likely provide novel insight into antibodies, track the modulation of TCR expression, and allow the monitoring lymphoid malignancies or possible detection of circulating tumor-infiltrating lymphocytes among other applications. Past attempts at recovering these repertoires have largely involved using polymerase chain reaction (PCR) amplification from cell populations followed by sequencing to detect each junction. Caveats include lack of chaining between alpha and beta chains and possible PCR amplification biases, although there have been some methods developed that attempt to address this [3–6].

The recent development of single-cell RNA sequencing (scRNAseq) allows the transcriptomes of thousands of cells to be processed simultaneously [7], bringing a way to identify subpopulations of cells and provide functional insights [8] such as the identification of each cell’s unique TCRs and paired alpha and beta heterodimers that were previously masked in the analysis of an ensemble of multiple cells. However, scRNAseq is not devoid of biases and noise. For example, scRNAseq can only quantify the expression of most highly expressed genes and likely suffers from PCR amplification biases.

Many current PCR-based methods for the amplification of V(D)J segments either use primer sets that introduce amplification artifacts owing to the differential amplification of some DNA templates over others, requiring the usage of complex normalization methods [9, 10] or require complex protocols based on template-switching effect of reverse transcriptase for the unbiased preparation of TCR cDNA libraries [6]. If successful, a single-cell sequencing model for TCRs may avoid these issues and recover these complex repertoires alongside the rest of the transcriptome present in T-cells.

In this paper, we address the accurate characterization of T-cell repertoires from scRNAseq data. We describe a computational method “single-cell TCRseq” (scTCRseq) to identify and count RNA reads mapping to specific TCR V and C region genes, then perform multiple alignment of reads mapping to V and C regions with a significant count to create consensus V and C gene sequence “contigs” across which gap-filling is performed in a manner similar to de novo transcript assembly. This allows the identification of a single cell’s V(D)J gene rearrangement(s) and recovers the entire receptor sequence including constant region and nucleotides inserted and deleted at junctions. We applied scTCRseq to identify paired alpha and beta receptor rearrangements by reanalyzing scRNAseq data from 91 naïve Cd4+ T helper cells in mice [11], RNAseq data generated from human Jurkat cell lines [12, 13], and also performed in silico simulations of single-cell T-cell RNAseq data. We found that scTCRseq facilitates the identification of productive and paired alpha and beta chain V(D)J TCR rearrangements and enables the recovery of full TCR including the nucleotide insertions and deletions at junctions in single T-cells. Single-cell TCRseq provides an avenue for phenotypic investigation of T-cells in conjunction with the accompanying whole-transcriptome data.

## Methods

### Single-cell TCRseq read filtering and V gene counting

Single-cell RNAseq reads were preprocessed in Trim Galore [14] using command line settings –stringency 5 and –q 20 and then the processed reads were formatted to FASTA format for processing by custom BLAST-mapping. The formatted reads were then submitted for nucleotide BLAST-mapping with a user-defined eValue against custom databases comprising TCR Alpha V genes, Beta V genes, Alpha C genes, and Beta C genes downloaded from IMGT [15], which had been processed to include only the first allele of each separate gene from the database, as the correct allele would subsequently be regained in the consensus alignment in the next stage of the pipeline. The BLAST expected value cutoff can be selected to be variable but parameter variation showed that a value of 1e-8 provided a good threshold to cut off spurious mappings and this was set as the default for the pipeline. This value is somewhat more stringent than the BLAST expected value threshold used to directly analyze CDR3 regions due to the smaller reference database used for this method and that scTCRseq is not aligning to the highly variable junctions that increase the uncertainty of mapping in these regions.

### TCR V gene consensus alignments

A table of the counts of reads meeting BLAST expected value cutoffs for each alpha and beta variable and constant genes was formulated for each sample. Due to the nature of the V and C genes in TCRs, some regions of these genes display high sequence similarity and a consequence of this was to produce ambiguity in the “top hit” produced in BLAST. Subsequent steps of reanalysis were required in the method to produce the correct candidate V and C region consensus sequences. For each candidate V or C region that had greater than 10 % of reads mapping to any V region mapping to the candidate in question, the following procedure was followed: (1) a pileup of reads mapping to this gene of interest was created; (2) if the pileup did not show coverage over the entire gene above a predetermined minimum of 5× then this gene was rejected as a false positive. Coverage of 5× was selected based on simulated data as sufficient to distinguish false-positive regions. False-positive hits showed apparent coverage in regions of high sequence similarity to the “true-positive” V or C genes and either zero or very low coverage in the regions where their sequence similarity diverged from the “true-positive” V or C genes; (3) for genes that met the minimal coverage over the entire gene the consensus sequence from the pileup was chosen as the candidate sequence; (4) candidate sequences for each of the loci (for example, if there were three consensus V beta sequences generated from candidates) were then compared to each other and if any sequence formed a subsequence to another it was removed from further analysis due to the two sets of reads mapping to the same gene; (5) this process was performed for the Alpha and Beta V and C regions for each candidate and a list of possible “contigs” was returned along with their mean coverage.

### Scaffold concatenation and gap-filling

Once a set of consensus V and C sequences was obtained for both the alpha and beta chains, they were then concatenated to produce gapped-scaffolds. If there were multiple V regions, then each V region was concatenated separately to the corresponding C region and a string of “N” nucleotides was generated in between the V and C gene consensus genes and put into a FASTA file for gap-filling to be performed. In detail, we ascertained the approximate length between V and C regions [16–18] and this distance was used to inform the gap between V and C regions, to produce concatenated V-C gap candidates with three nucleotides trimmed from the ends of the V and C consensus sequences. As required by GapFiller, a library file was then created for each sample containing the gapped contig fasta file, the processed reads files, with bwa [19] chosen for the alignment algorithm stage for aligning reads with the gapped contig. An estimated insert size was calculated based upon the mean length of the reference J or D-J regions with a tolerance of plus or minus 50 %, resulting in an insert estimate of 33–99 bp which was easily sufficient to encompass any variation in CDR3 lengths that had previously been observed in TCR alpha or beta junctions [20–22]. GapFiller [23] was then run on these libraries with parameters (–r 0.7 –n 5 –d 50 –t 0 –g 2 –T 1 –i 3) and the minimum number of reads needed to call a base during an extension set to reflect the minimum coverage selected earlier and the minimum number of overlapping bases with the sequences around the gap set be 20, unless for very short read lengths where it was set lower.

A junction was considered to be filled if the program successfully closed the junction gap fully, otherwise the gapped sequence was rejected. Output files were then created containing all full closed gap receptor sequences and also the candidate V and C consensus sequences along with their mean coverage.

Source code written in python is available at https://github.com/ElementoLab/scTCRseq together with documentation on how to install and run the pipeline.

### RNA sequencing data

Single-cell RNAseq data for the mouse the T helper cells have previously been described [11] and we downloaded data from the ArrayExpress [24] under accession number E-MTAB-2512. The RNAseq data generated for three replicate human Jurkat cell lines was downloaded from GEO [12] under accession number GSE45428. Simulated RNAseq reads for the in silico testing was generated as follows: GemSim [25], a General Error-Model based SIMulator, capable of generating paired-end reads generated using a reference genome and a given error model was used on the hg19 human reference genome to generate read pairs of read lengths 25 bp, 50 bp, 75 bp, and 100 bp with a mean length of fragments of 300 bp with standard deviation of 30 bp. Noise and errors were added according to the ill100v4_p error model: llumina GA IIx with Illumina Sequencing Kit v4 chemistry, paired reads. TCR alpha and beta chain sequences were generated for 30 pairs by randomly concatenating variable (diversity), junction, and constant regions (V-J-C for alpha and V-D-J-C for beta) selected at random from the IMGT reference human databases for each gene region [15]. Random bases were then inserted at the junction segments of lengths 0 bp, 3 bp, 6 bp, and 9 bp to model the junctional diversity of VDJ recombination that occurs in TCRs. Empirically we calculated from the Jurkat cell line RNAseq data that approximately 0.3 % of all mapped reads mapped to the reconstructed TCR loci; therefore, for each simulated alpha and beta chain sequence we then again applied GemSim to the synthetic TCR alpha and beta sequences to generate simulated read pairs at the required length and error model and concatenated them with the reference RNAseq simulated data from the hg19 models in a ratio of 3:997 to generate realistic error-rich read-pair data generally reflective of a scRNAseq run at the required read length and depth, with the depths selected so that on average the TCR alpha and beta chains had average coverage of 10×, 50×, and 100×, generating 120 simulated paired read files for each read length and average TCR coverage.

### Transcript identification and quantification

Paired-end reads from the 91 single-cell RNAseq samples were mapped simultaneously to the Mus musculus genome (Ensembl version 38.70) and we counted reads for each gene using htseq-count [26] to generate a raw counts table of genes across all 91 samples. The raw counts were then processed and filtered according to the edgeR vignette [27] and the unadjusted FPKM for each gene was calculated as its cpms (counts per million (CPM) mapped reads) normalized by the summed length of its exons.

### Data quality control and pre-filtering

Paired-end reads from the 91 single-cell RNAseq samples were mapped simultaneously to a custom reference genome comprising the Mus musculus genome (Ensembl version 38.70) along with the ERCC spike-in sequences (available from https://www.ebi.ac.uk/arrayexpress/experiments/E-MTAB-2512/files/). After alignment was performed, we counted reads for each using htseq-count [26] to generate a raw counts table of genes and reads mapping to ERCC spike-ins across all 91 samples (Additional file 1: Table S1). Following the counting of mapped reads, we applied the following quality control filters: total number of reads > 0.25 million, percentage of reads mapping to known exons > 40 %, number of genes detected per cell > 6000 and ratio of reads mapping to ERCC < 0.6. This produced 71 samples meeting these criteria.

## Results

### A pipeline for TCR analysis of scRNAseq

**Fig. 1:**
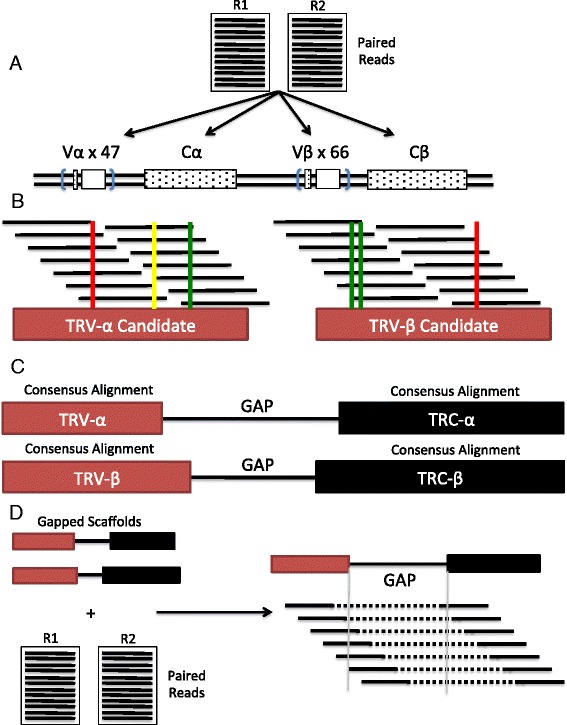
*Schematic overview* of scTCRseq pipeline. **a** Input fastq files are BLAST-aligned to custom reference TCR alpha and beta variable and constant regions genes. **b** Consensus-driven multiple alignment to candidate genes is performed to create V and C gene sequence “contigs.” Correct alleles are inferred from the consensus. **c** Each consensus V region is concatenated to its corresponding C region consensus with a gap to create a scaffold to perform gap-filling. **d** GapFiller finds read pairs of which one member matches within a sequence region and the second member falls (partially) within the gap to join the scaffolds and recover full alpha or beta chains if successful

The scTCRseq pipeline takes as input unaligned single or paired-end reads from whole transcript scRNAseq. It is run on each cell individually. Briefly, the pipeline takes paired-end reads as input and first creates high-confidence V and C region sequences separated by a gap, then fills the gap using a process similar to de novo assembly. In more detail, the pipeline works as follows (Fig. 1): first, it maps reads using NCBI nucleotide BLAST against a custom blastn [28] database comprising TRA/B variable and constant gene loci downloaded from IMGT [29] (Fig. 1a). For each set of reads mapping to the V region alpha or beta chains, we then perform a pileup and multiple alignment of these reads to any particular variable gene that shows a significant number of mapped reads (we set the threshold at 10 % of mapped reads but this can be altered in the pipeline) to generate consensus variable gene sequences that satisfy a minimum coverage (Fig. 1b). A multiple sequence alignment is also performed on the set of reads mapping to constant regions to generate consensus constant region sequences.

Once a set of V and C region “contigs” are obtained for variable and constant regions of interest in the single-cell sample, each consensus V region is concatenated to its corresponding C region consensus with a gap inserted between the V and C regions to create a scaffold (Fig. 1c). The gap is filled using gap-filling software GapFiller [23]. To achieve this, GapFiller uses all paired reads from the sample and seeks to find read pairs for which one read matches within a sequence region and the second read falls (partially) within the gap (Fig. 1d). The latter reads are then used to close the gap through a predefined sequence (*k-mer*) overlap. If a gapped scaffold satisfies the gap-filling procedure at a predefined minimum coverage they can then be analyzed for V(D)J sequence alignment in tools such as igBlast [30] or IMGT V-QUEST [31]. A full account of the pipeline is also provided in the “Methods” section.

The method outlined offers a novel approach to the recovery of TCRs in scRNAseq data. It differs from currently available software such as MiTCR [32] and Vidjil [33] in that scTCRseq adopts an “outside-in” rather than an “inside-out” approach. Alternative software is designed primarily to analyze CDR3-containing reads generated from primer and PCR-based approaches; in general, they target the extraction of the entire CDR3 sequence and then report on clonotypes based upon the CDR3 frequency without trying to accurately determine the specific V or C gene unless very long read coverage is available.

scTCRseq is built with the presumption that the RNAseq data came from a single cell with at most two or three separate alpha or beta chains to be identified and therefore takes a different approach. The first step of scTCRseq is to determine with high accuracy the sequences of the variable and constant regions of an alpha or beta chain and generate two separate “contigs” (a full variable region and a full constant region of the chain in question) with the aim of leveraging this information to join the “contigs” together using a gap-filling assembly akin to some methods used in de novo transcript assembly [34, 35].

There are multiple reasons why we have adopted this different approach: due to the CDR3 sequence being inherently more variable than the V and C segments either side of it, the alignment stage for software that detects CDR3 sequences relies upon generating vastly larger reference libraries of all possible junction sequences with the additional problem of accounting for nucleotide insertions and deletions at the junctions. This means that the alignment for scTCRseq in the V and C regions can be more stringent and then use a consensus-driven approach to generate the V and C regions that is more robust both to shorter read lengths and higher sequencing error-rates. scTCRseq takes advantage of the information inherent in the read pairs (given sufficient information about the total read length) so that we can leverage a gap-filling assembly as we have high confidence of the V and C regions either side of the gap, unlike the “inside-out” approaches that necessarily require reads to span the entire length of the CDR3 region and take no advantage of information of the mate-pair read. This means that scTCRseq can perform at lower read length and lower read depth, as we use the information of a far higher proportion of RNAseq reads mapping to a TCR to provide information either regarding generating the consensus V and C “contigs” or in assembling across the gap between the two.

Unlike many cases of de novo transcript assembly we know the range of the gap size between “contigs” with high accuracy from analysis of currently known CDR3 lengths [16–18]. The assembly is also agnostic to generating a library of junction sequences and instead uses a consensus approach, which allows reads that do not quite span the whole CDR3 region to provide additional information to the generation of either the alpha or beta chain sequence.

### Validation and in silico simulations

To validate our method for the recovery of full-length TCR alpha and beta chains, we ran scTCRseq on RNAseq data generated from a human Jurkat cell line [12, 13] for which the complete alpha and beta receptor sequences had previously been determined [36, 37]. RNAseq data from the Jurkat cell line provides a good control to scRNAseq data due to its clonal nature and because it has been extensively studied the sequence of it alpha and beta chain TCRs are well known [38]. The Jurkat T-cell messenger RNA was then analyzed on an Illumina HiSeq2000 with each run producing approximately 80 million reads with paired-end read length of 101 bp and total length of approximately 350 bp using the Illumina TruSeq RNA Sample Prep Rev. A.

First, we asked whether scTCRseq can recover the alpha and beta chains at different total read depths observed in the three replicate samples. We also compared the performance of the pipeline on the Jurkat cell line data at various read depths with two other programs: iSSAKE [39] and VIDJIL [33]. These programs had previously been designed to process CDR3-containing reads in RNAseq libraries.

The scTCRseq sequencing pipeline was run on the three replicate samples with standard parameters (with BLAST eValue threshold 1e-8, minimum supporting read coverage 5 and estimated mean insert size of 148) and in all three cases returned one productive alpha and beta chain that were identical (alpha chain of TRAV8-4*01/TRAJ3*01/TRAC*01 and beta chain of TRBV12-3*01/TRBD1*01/TRBJ1-2*01/TRBC2*01) and which had VJ and V(D)J combinations in agreement with the reference sequences (Additional file 2: Table S2). This confirms that scTCRseq can reliably recover alpha and beta chain sequences from a validated sample. We also ran the programs iSSAKE and VIDJIL on these datasets with a range of parameters appropriate for the RNAseq dataset (iSSAKE –m 15,30,45,60 –o 2,5,10,100 –r 0.7 –p 1 and a range of values for –d from 100 to 400 and VIDJIL –c clones –r 5,50,100). In the case of ISSAKE, we were unable to produce any clonotypes that mapped to a TCR alpha or beta chain sequence or produced a correct result when subsequently analyzed using IMGT V-Quest [31] while trying all different combinations parameters listed. VIDJIL aligns reads spanning the junctions and therefore was not able to return the entire sequence of either the alpha or beta chain and was unable to specify exactly which variable genes were the correct assignment in either case (the short length of V region sequence resulted in five possible beta variable V regions and three possible alpha variable V regions). However, VIDJIL was able to extract a correct clonotype corresponding to both alpha and beta chains for the TCR and also amino acid sequences for the junctions that were in agreement with scTCRseq.

We performed subsampling of the read-sets to determine an approximate read coverage required to infer accurate detection of the entire alpha and beta chains, and again compared this to the read depth required for the program VIDJIL to accurately infer the junction sequences. We used the program seqtk (downloaded from https://github.com/lh3/seqtk) to perform subsampling on the three replicate sets of Jurkat cells, subsampling at depths of 50 k, 100 k, 500 k, 1 million, and 5 million read pairs and used six different random seeds for each sample and coverage to produce 18 independent read-sets at each depth. We ran scTCRseq and VIDJIL with their standard parameters (scTCRseq with BLAST eValue threshold 1e-8, minimum supporting read coverage 5, and estimated mean insert size of 148 and VIDJIL: -c clones –r 5) and the results (Additional file 2: Table S2) show that scTCRseq recovered the beta chain sequence in all cases at a depth of 100 k read pairs and the alpha chain in all cases at a read depth of 500 k read pairs. When VIDJIL was run for the same datasets the beta chain required 500 k read pairs to discover the junctions in all cases and the alpha chains required 1 million read pairs to discover the junctions in all cases. Again, in these cases VIDJIL was unable to identify the correct variable genes due to only being able to align reads spanning the junctions.

To further validate scTCRseq’s ability to filter and accurately determine the TCR alpha and beta chain sequences, we applied it to a set of error-prone simulated reads generated at various read lengths and read counts. GemSim [25], a General Error-Model based SIMulator, capable of generating paired-end reads generated using a reference genome and a given error model was used on the hg19 human reference genome to generate read pairs of read lengths 25 bp, 50 bp, 75 bp, and 100 bp with a mean length of fragments of 300 bp with standard deviation of 30 bp. Noise and errors were added according to the ill100v4_p error model: llumina GA IIx with Illumina Sequencing Kit v4 chemistry, paired reads. TCR alpha and beta chain sequences were generated for 30 pairs by randomly concatenating variable (diversity), junction, and constant regions (V-J-C for alpha and V-D-J-C for beta) selected at random from the IMGT reference human databases for each gene region [15]. Random bases were then inserted at the junction segments of lengths 0 bp, 3 bp, 6 bp, and 9 bp to model the junctional diversity of VDJ recombination that occurs in TCRs. Empirically, we calculated from the Jurkat cell line RNAseq data that approximately 0.3 % of all mapped reads mapped to the reconstructed TCR loci. Therefore, for each simulated alpha and beta chain sequence we then again applied GemSim to the synthetic TCR alpha and beta sequences to generate simulated read pairs at the required length and error model and concatenated them with the reference RNAseq simulated data from the hg19 models in a ratio of 3:997. This generates realistic error-containing read-pair data generally reflective of a scRNAseq run at the required read length and depth. In these random runs, the depths were selected so that on average the TCR alpha and beta chains had average coverage of 10×, 50×, and 100×, generating 120 simulated paired read files for each read length and average TCR coverage. We defined that scTCRseq had accurately recovered the alpha or beta chain sequence in question if it fully recovered the junction sequence and sufficient V, J, and C gene to uniquely determine the chain that was simulated. We also ran VIDJIL on the simulated data to check the recovery rate of the junction sections. The results of the simulations (Additional file 3: Table S3) show that given sufficient coverage scTCRseq was able to accurately recover both TCR loci at all read length at greater than or equal to 90 % accuracy for TCR average coverage of 50×. Due to the requirement to have reads spanning the entire junctional segments of the alpha and beta chains, VIDJIL was unable to determine any alpha or beta chain sequence for simulated data with read lengths 25 bp or 50 bp, but could accurately determine the junction sequence at rates comparable to scTCRseq for coverage of 50× or greater (without determining the unique correct V genes). However, scTCRseq was able to accurately determine the TCR loci at lower coverage, especially for longer read lengths where at 10× coverage and 100 bp read length it recovered 94 % of alpha chain sequences and 100 % of beta chain sequences versus 48 % for VIDJIL in both cases.

Altogether, the results from running scTCRseq on the Jurkat cell lines and simulated single-cell data indicate that this method can accurately determine entire TCR loci from single T-cell sequence data, with a greater sensitivity than other current methods and at a greater range of read lengths.

### Application to single T-cell RNAseq shows that TCR variable region transcripts are highly expressed

We applied the method to study two populations of differentiating T helper cells that had been profiled using scRNAseq previously [11]. In brief, the naive Cd4^+^ T helper cells were activated and polarized with IL-4 to induce differentiation towards a T_H_2 subtype. Four and a half days after stimulation, the cells were sorted into a G4P group (fourth generation, IL-13–GFP^+^ cells) and a G2N group (second generation, IL-13–GFP^−^ cells) and pooled in equal proportions. A set of 91 asynchronously dividing cells from the pool (including both fully and partially differentiated cells) was captured using the Fluidigm C1 system and sequencing libraries were prepared and processed, producing libraries of paired-end 75 bp reads with an average of approximately 16 million paired end reads (mean 16.06, minimum 2.20, maximum 28.85, standard deviation 4.62 million reads) for each single-cell sample, a summary of read counts. Further information on the protocol and libraries are available at [40, 41] and a summary of read-count, quality statistics, and phenotypes for each sample is shown in Additional file 1: Table S1.

**Fig. 2:**
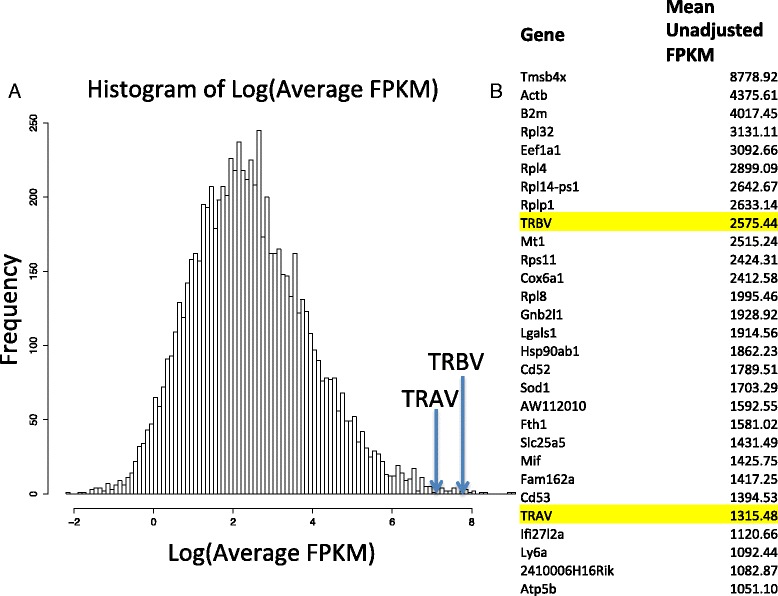
Expression of TCR alpha and beta chain variable gene transcripts relative to the rest of the transcriptome. **a** *Histogram* of the logarithm of the mean unadjusted fragments per kilobase per million mapped reads (FPKMs) over all 91 mice T-cell samples, comprising average FPKMs of 7990 genes. The location of the average logarithm FPKMs of the TRAV and TRBV genes is also outlined. **b** *List* of the top 30 mean-expressed genes over all 91 mice T-cell samples comprising average FPKMs of 7990 genes with the location of TRAV and TRBV genes on the list

After the read-data were downloaded from ArrayExpress [24] under accession number E-MTAB-2512, we compared the counts of reads mapping to the TCR variable genes to the transcriptome as a whole to gauge relative expression of these genes. After mapping to the reference genome, the raw counts were normalized and their counts per million reads were calculated in the manner of the edgeR package [27]. We observed that the TRAV and TRBV FPKM (fragments per kilobase of exon per million reads mapped) values were, on average across all the samples, some of the most highly expressed of the 7990 genes observed after quality control (Fig. 2a). The list of most highly expressed gene transcripts is seen in Fig. 2b. Among the most highly expressed genes were B2m (a component of MHC class I molecules), Actb (a commonly used housekeeping gene in T lymphocytes) [42], multiple ribosomal proteins, and Mif (a key regulator in innate immunity) [43].

### Single-cell TCRseq recovers paired expression of TCR alpha and beta chain variable genes

Quality control was performed (“Methods” and Additional file 1: Table S1) and 71 samples were subsequently selected for processing in scTCRseq (with BLAST eValue threshold 1e-8, minimum supporting read coverage 5, and estimated mean insert size of 175). The reads were mapped in BLAST using the custom mice variable and constant gene databases as described previously. The TCR variable region counts for each sample were counted and analyzed (Additional file 4: Table S4). The tables produced are raw counts of how many reads mapped to a particular alpha or beta variable gene in each sample. For each sample, the relative frequency of reads mapping to a particular variable gene were then calculated and for the beta variable genes the single variable gene with the highest percentage mapping reads had a mean of approximately 84 % and median of approximately 89 % of the sum of all reads that mapped to any beta variable genes for the sample. For the alpha variable genes, the corresponding figures were a mean of approximately 54 % and median of 52 %, this lower number could be explained in a number of ways: either by the increased sequence similarity of the alpha chain variable genes making a precise mapping more difficult to achieve and also that in many cases it is possible for a single T-cell to have multiple distinct alpha or beta chains [3, 44]. In the case where multiple variable regions of a particular chain had a significant count, they were all consensus aligned, which either resolved the two sequences to be identical or produced multiple candidate sequences for gap-filling assembly.

**Fig. 3:**
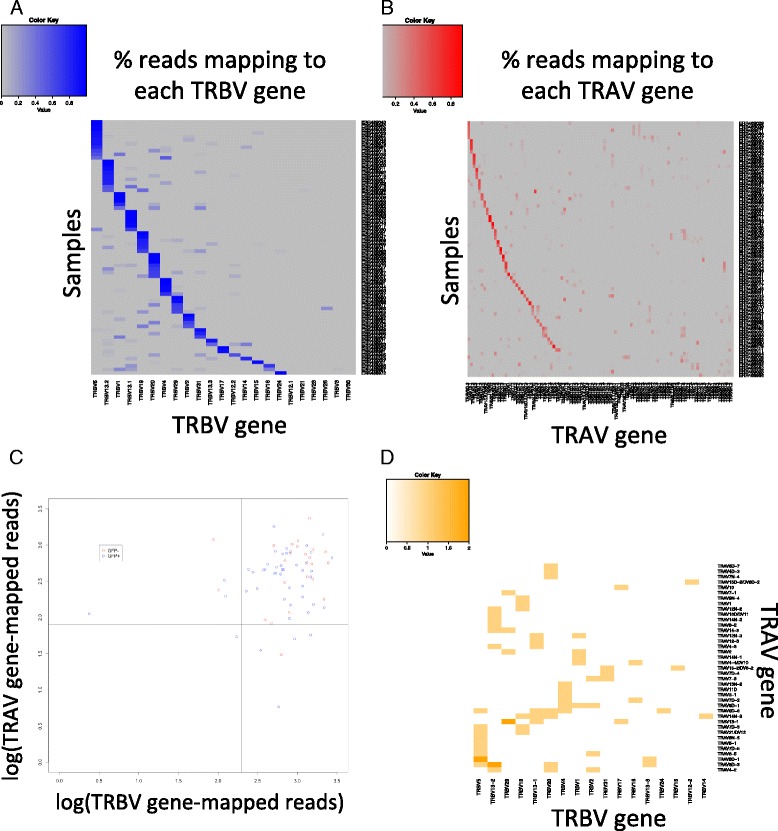
Count-based analysis of TCR alpha and beta chain variable gene transcripts across the filtered 71 mice T-cell samples. **a** *Heatmap* of percentage of nucleotide BLAST-mapped TRBV genes mapping to individual TRBV genes (*x-axis*) across 71 mouse T-cell samples (*y-axis*). Increasing shades of *blue* denote increased percentage of all TRBV-mapping reads mapping to a particular TRBV gene. **b** *Heatmap* of percentage of nucleotide BLAST-mapped TRAV genes mapping to individual TRAV genes (*x-axis*) across 71 mouse T-cell samples (*y-axis*). Increasing shades of *red* denote increased percentage of all TRAV-mapping reads mapping to a particular TRAV gene. **c** *Log-log plot* of total reads that nucleotide BLAST-mapped to TCR variable regions. The *x-axis* measures the logarithm of the count of total reads mapping to any TRBV gene for a single sample and the *y-axis* measures the logarithm of the count of total reads mapping to any TRAV gene. Each *colored point* represents an individual mouse T-cell samples with *read points* denoting GFP negative T-cells and *blue points* denoting GFP positive T-cells. The *lines* at x = 2.3 and y = 1.9 represent cutoffs for which samples were selected for further analysis based upon their expression of TCR transcripts (bottom 10th percentile cutoff). The 59 samples in the *upper right quadrant* of the figure underwent further targeted assembly. **d** *Heatmap* of paired TCR alpha and beta chain expression with *x-axis* representing chosen TCR beta chain V usage and the *y-axis* representing TCR alpha chain V usage. *Color scale* ranged from *white* for no particular (TRBV, TRAV) usage to *dark orange* for two samples observing the same (TRBV, TRAV) usage

We observed preferential expression of several TCR beta variable genes (TRBV5,20,13-1,1-9,1,13-2,4,29) (Fig. 3a) but a more diverse usage of the alpha variable genes across samples (Fig. 3b) which has been observed in previous studies [45]. The relative expression of the TCR variable genes varied widely across the 71 samples (Fig. 3c) where the reported total normalized cpms for the alpha and beta chain variable TCR genes with the TRBV genes showed a wide range.

Paired analysis in the 48 samples of the major alpha and beta variable genes (Fig. 3d) showed that the combined recovered alpha and beta paired variable genes was diverse in terms of the count of alpha-beta chain pairings, with the same alpha-beta chain pairing occurring twice only three times. Although this is a single set of sample observations, it shows that this single-cell method could provide a rich analysis of the TCR alpha-beta pairing and give further insights into the repertoire diversity under various adaptive immune conditions, and whether these chains are always selected together independently [46].

After normalizing the total reads mapped to TRAV and TRBV genes by the total number of aligned reads in each sample, we found there was a correlation of the log-counts per million reads between TRAV and TRBV expression of 0.45 (R^2^ = 0.23, *p* = 1e-06) and a wide variability in the log-normalized density of reads (TRAV: mean, 2.50; standard deviation, 0.56; TRBV: mean, 2.80; standard deviation, 0.52) among the 71 samples. There was no significant difference in expression of TCR variable genes among the two cell phenotypes of GFP-positive and GFP-negative cells (briefly naïve Th cells from IL-13-eGFP mice had been activated under conditions inducing Th2 differentiation; IL-13-eGFP-negative, undivided cells and IL-13-eGFP-positive cells that had undergone four cycles of cell division) under a *t*-test (TRAV: *p* = 0.36; TRBV: *p* = 0.33).

After selection, the potential alpha and beta chains were concatenated and gap-filled assembly was performed. The consensus alignment produced at least one alpha chain V sequence in 65 samples (91.5 %) and at least one beta chain V sequence in 69 samples (97.2 %) that were then used in gap-filling assembly. The assembled transcripts were also analyzed in IMGT V-QUEST [31] producing V, D, and J region alleles, as well as percentage identity and nucleotide insertions/deletions at junctions (Additional file 5: Table S5). At least one productive alpha chain was recovered in 77.5 % of samples and at least one beta chain was recovered in 91.5 % of samples. The recovery rate of functional TCR chains we demonstrate is within the range of what has been achieved by PCR-based methods [1, 3, 47, 48]. For samples that showed high expression of both TRAV and TRBV genes (Additional file 1: Table S1), the recovery rate for at least one productive alpha chain was 88.1 % and for beta chains 96.6 %. GFP-positive cells had a recovery rate of 73.9 % alpha chains and 95.6 % beta chains; GFP-negative had a recovery rate of 84 % alpha chains and 84 % for beta chains.

Multiple alpha and beta chains were also recovered for a single cell: two productive alpha chains were recovered in 20 % of cells that recovered at least one alpha chain and two productive beta chains were recovered in 23.1 % of cells that recovered at least one productive beta chain. We also observed two samples in which three productive alpha chains were recovered (in one of these samples, there were two productive beta chains recovered so the possibility of there being multiple cells present in this sample cannot be discounted). These rates of recovery for multiple TCR alpha and beta chains are within the range of previously reported studies that have reported that up to 42 % of T-cells contain two alpha chain recombinants and up to 22 % contain two beta chain recombinants [44, 49, 50].

**Fig. 4:**
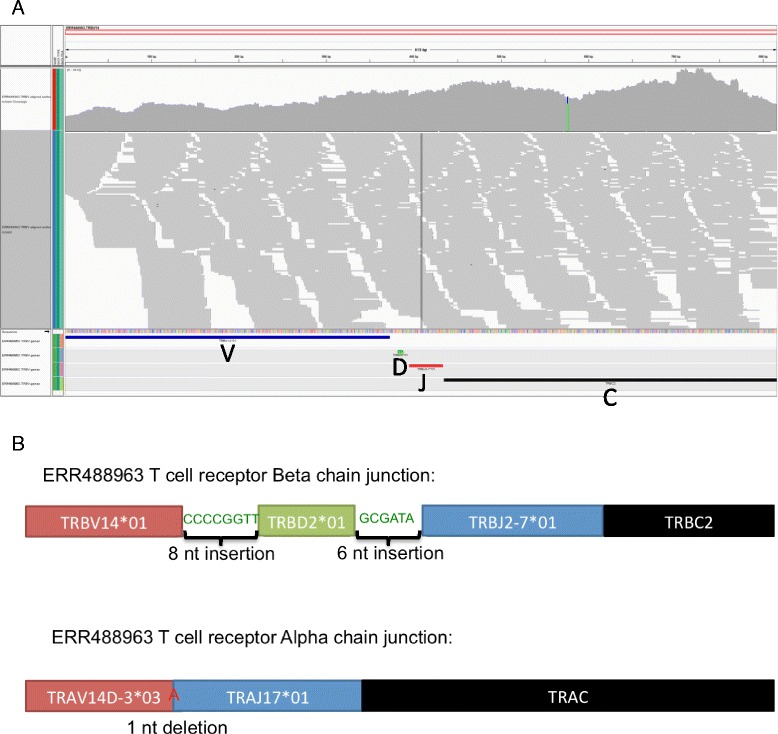
TCR V(D)J sequence usage in Sample ERR488963. **a** *IGV plot* of read coverage for entire assembled TCR beta chain sequence. The *tracks* underneath the reads denote the TCR beta V gene (TRBV14*01 in *blue*, TRBD2*01 in *green*, TRBJ2-7*01 in *red*, and TRBC2 in *black*). **b** *Schematic* of nucleotide insertions and deletions at V(D)J junctions in both alpha and beta chains

An example (Fig. 4a) of a full productive beta chain V(D)J rearrangement recovered from a sample is shown along with the reads mapping to this receptor sequence as a reference with mean coverage of 1857× per bp. The full sequence also allowed the elucidation of the productive junctional diversity regions (Fig. 4b) in both alpha and beta chains.

## Discussion

The ability to recover the entire sequence of TCRs is critical as these receptors play an essential role in controlling the selection, function and activation of T-cells [51]. In this paper, we report the development of a novel pipeline for the analysis of scRNAseq data with the purpose of the recovery of the entire paired alpha and beta chain of the TCR of a single cell. The possibility of identifying both the TCR alpha chain paired with a TCR beta chain also provides a more unique CDR3 alpha/beta signature for tracking which has many possible applications across the fields of adaptive immunity including vaccination development and response [52], clone tracking [53], and immunotherapy [54].

Current methods to extract information on TCR usage in cells require the extensive usage of primers and a subsequent PCR amplification that result in the risks of primer bias, misamplification or even the failure of amplification [55]. Our new framework scTCRseq avoids these problems by addressing TCR usage at a single-cell level while also providing paired alpha and beta chain usage and more complete sequence information as PCR-amplified transcripts are necessarily shorter than the true transcript. Also the extraction of TCR sequence information from scRNAseq avoids the costs of performing another costly and involved laboratory procedure.

In this study, we outlined a new method for characterizing the TCR alpha and TCR beta chains expressed by T-cells and as a proof of concept applied it to a set of 71 single-cell mouse T-cell RNAseq samples. We showed that the transcript expression of these genes are among the most expressed in T-cells (Fig. 2), demonstrated the identification of the TCR alpha and beta variable gene usage across each sample along with their paired usage (Fig. 3), and in a majority of cases were able to assemble the entire alpha and beta chain. As a validation we applied the pipeline to recover of full-length TCR alpha and beta chains in human Jurkat cell lines [12, 13] for which the alpha and beta receptor sequences had previously been determined [36, 37] (Additional file 2: Table S2) and also tested the performance of the pipeline on a set of simulated read datasets to assess its sensitivity and accuracy, while also comparing it to other programs currently available that detect CDR3 sequences (Additional file 3: Table S3). We were also able to demonstrate that the recovery of full TCR sequences is possible at a number of different read lengths, and gauge a minimum read depth or sequencing coverage at which this is achievable. Altogether, these results demonstrate that paired alpha and beta chain recovery is feasible from scRNAseq. This opens up interesting avenues in studies of immune response to a variety of challenges as well as in immunotherapy to explore clonal dynamics before and after treatment and the determinants of clinical response to checkpoint blockade. Potential other directions for further research include adaptation of scTCRseq to other sequencing technologies such as DropSeq [56].

## Abbreviations

BLAST, basic local alignment search tool; cDNA, complementary DNA; Cpms, counts per million mapped reads; G2N, second generation IL-13–GFP^−^ cells; G4P, fourth generation IL-13–GFP^+^ cells; IMGT, the international immunogenetics information system; PCR, polymerase chain reaction; RNA, ribonucleic acid; RNAseq, RNA sequencing; scRNAseq, single-cell RNAseq; scTCRseq, single-cell T-cell receptor sequencing; TCR, T-cell receptor; TRA, T-cell receptor alpha locus; TRB, T-cell receptor beta locus; V(D)J, variable, joining, and in some cases, diversity gene segments

## Additional files

Additional file 1: Table S1. Summary data of 91 single-cell mouse T-cell RNAseq samples. Provided as a separate Excel file. (XLSX 59 kb) Additional file 2: Table S2. Results of scTCRseq and VIDJIL run on three replicate Jurkat T-cell RNAseq samples. Provided as a separate Excel file. (XLSX 14 kb) Additional file 3: Table S3. Results of scTCRseq and VIDJIL run on simulated scRNAseq data at different read lengths and coverage. Provided as a separate Excel file. (XLSX 10 kb) Additional file 4: Table S4. Raw counts of reads mapping to TRA and TRB variable gene loci in 71 selected single-cell mouse T-cell RNAseq samples. Provided as a separate Excel file. (XLSX 69 kb) Additional file 5: Table S5. Summary of recovered alpha and beta chains in 71 selected single-cell mouse T-cell RNAseq samples. Provided as a separate Excel file. Samples with multiple productive alpha or beta chain sequences highlighted in yellow. (XLSX 96 kb)
